# Characteristics of parents accessing a new postmortem imaging service to diagnose miscarriage in the United Kingdom

**DOI:** 10.1016/j.xagr.2025.100573

**Published:** 2025-10-03

**Authors:** Holly Ellard, Celine Lewis, Ulrika Kreicbergs, Audrey Lamouroux, Owen J. Arthurs, Ian C. Simcock

**Affiliations:** Department of Population, Policy and Practice, University College London Great Ormond Street Institute of Child Health, London, England, United Kingdom; Department of Obstetrical Gynecology, Nîmes University Hospital, Nimes, France; Fetopathology Unit, Department of Clinical Genetics, University Hospital of Montpellier, Montpellier, France; Joint Research Unit 5221 between the National Center for Scientific Research and the University of Montpellier, BioNanoImaging Foundry User Facility Imaging, Charles Coulomb Laboratory, Laboratoire d'Informatique, de Robotique et de Microélectronique de Montpellier, Centre National de la Recherche Scientifique, University of Montpellier, Montpellier, France; Department of Population, Policy and Practice, University College London Great Ormond Street Institute of Child Health, London, England, United Kingdom; Department of Clinical Radiology, Great Ormond Street Hospital for Children, London, England, United Kingdom; Great Ormond Street Hospital National Institute for Health and Care Research Biomedical Research Centre, London, United Kingdom

I.C.S. is funded through the National Institutes of Health Research (NIHR) for Patient Benefit (grant number: NIHR206174), NIHR Development and Skills Enhancement Award (grant number: NIHR302390), and NIHR Clinical Doctoral Research Fellowship (grant number: ICA-CDRF-2017-03-53). O.J.A. is funded through the NIHR Career Development Fellowship (grant number: CDF-2017-10-037), and C.L. is funded through the NIHR Advanced Fellowship (grant number: NIHR-300099). The views expressed are those of the authors and not necessarily those of the National Health Service, the NIHR, or the Department of Health and Social Care. The funder had no role in the study design, data collection, data analysis, data interpretation, or writing of the report.

## OBJECTIVE

Postmortem imaging is a feasible and acceptable noninvasive alternative to conventional perinatal autopsy that is predicted to increase uptake.^1^ Since 2016, microfocus computed tomography (micro-CT) postmortem imaging has been offered to parents at Great Ormond Street Hospital (GOSH) to investigate early pregnancy loss (<24 weeks) with high rates of uptake and diagnostic accuracy.^2^^,^^3^ Micro-CT has been shown to detect an abnormality in 31% of cases and, together with placental examination, provides sufficient diagnostic information to avoid an invasive autopsy in 96% of cases.^2^^,^^3^ In this retrospective audit of practice, we describe the characteristics of parents who accepted or declined micro-CT as part of a wider mixed methods evaluation of the new service to guide future service development.

## STUDY DESIGN

Clinical and demographic details of all perinatal deaths referred for micro-CT postmortem investigation at GOSH, London, United Kingdom, between 2015 and 2023 were extracted from health records, including parental characteristics (age, ethnicity, and obstetric history) and referral maternity unit. obstetric history was described in terms of number of previous pregnancies and number of previous miscarriages or terminations. The parents who chose to opt out through the National Data Opt-Out were excluded from the study.

## RESULTS

During the 8-year study period, 1000 parents were offered micro-CT, of which 986 of 1000 (99%) consented and 14 of 1000 (1%) declined (Table). Referrals were received from 13 different National Health Service trusts, with 3 trusts responsible for approximately half of all referrals. Among parents who consented, the most common ethnic background of parents was White (45% of mothers and 43% of fathers), and most mothers were under the age of 35 (70%) with no previous history of pregnancy loss (56%).

**Table:** Characteristics of parents who accepted or declined postmortem micro-computed tomography imaging

	Accepted		Declined		
Demographic	Frequency (n=986)	Percentage	Frequency (n=14)	Percentage	Percentage of the demographic group that declined
Ethnicity					
Mothers					
Asian	91	9%	0	0%	0%
Black	102	10%	5	36%	5%
White	445	45%	5	36%	1%
Other	37	4%	2	14%	5%
No data	311	32%	2	14%	1%
Fathers					
Asian	114	12%	—	—	—
Black	93	9%	—	—	—
White	428	43%	—	—	—
Other	27	3%	—	—	—
No data	324	33%	14	100%	4%
Age at referral					
Mothers					
16–25 y	122	12%	1	7%	1%
26–35 y	571	58%	8	57%	1%
≥36 y	281	29%	5	36%	2%
No data	12	1%	0	0%	0%
Mean±SD (range)	32±6 (16–53)		33±5 (25–44)	—	—
Fathers					
16–25 y	21	2%	—	—	—
26–35 y	157	16%	—	—	—
≥36 y	153	16%	—	—	—
No data	655	66%	14	100%	2%
Mean±SD (range)	36±7 (18–58)	—	—	—	—
obstetric history					
Number of previous pregnancies					
0	287	29%	3	21%	1%
1	229	23%	4	29%	2%
2	177	18%	4	29%	2%
≥3	273	28%	3	21%	1%
No data	20	2%	0	0%	0%
Median (range)	2 (0–13)	—	3 (0–7)	—	—
Number of previous miscarriages or terminations					
0	549	56%	9	64%	2%
1	199	20%	3	21%	1%
2	110	11%	2	14%	2%
≥3	105	11%	0	0%	0%
No data	23	2%	0	0%	0%
Median (range)	0 (0–8)	—	0 (0–2)	—	—

*SD*, standard deviation.

## CONCLUSION

Given that established risk factors for miscarriage include Black ethnicity, female age >35 years, and a history of recurrent miscarriage,^4^ questions remain about why those groups constituted a small proportion of mothers accessing the micro-CT service (10% of mothers were Black, 29% were aged >35 years, and 11% had ≥3 previous miscarriages or terminations). Unlike rates of stillbirth and perinatal deaths at >24 weeks of gestation, national data on miscarriage are not collected to compare our study population against.^5^ Therefore, it is not possible to know whether our study population proportions are representative of the population affected by early miscarriage or whether, and perhaps more likely, they reflect disparities in service access and insufficient reach to underserved communities. In addition, this study highlighted that less demographic data are routinely collected about fathers compared with mothers. These data should be collected with the same frequency and detail to avoid gender disparities in miscarriage research.

The next step for our mixed methods evaluation is to explore the perceptions and experiences of parents and health professionals accessing the postmortem micro-CT imaging service at GOSH and the parents who declined a micro-CT investigation through surveys and interviews. By triangulating our findings, we aim to highlight the factors and processes that underpin current service provision and parental decision-making and to understand if and why disparities in access exist.

## CRediT authorship contribution statement

**Holly Ellard:** Writing – review & editing, Writing – original draft, Visualization, Project administration, Formal analysis. **Celine Lewis:** Writing – review & editing, Supervision, Project administration, Methodology, Investigation, Funding acquisition, Conceptualization. **Ulrika Kreicbergs:** Writing – review & editing, Supervision. **Audrey Lamouroux:** Project administration, Formal analysis, Data curation. **Owen J. Arthurs:** Writing – review & editing, Supervision, Methodology, Funding acquisition, Conceptualization. **Ian C. Simcock:** Writing – review & editing, Supervision, Resources, Project administration, Methodology, Investigation, Funding acquisition, Formal analysis, Data curation, Conceptualization.
